# Effects of Dielectric Barrier on Water Activation and Phosphorus Compound Digestion in Gas–Liquid Discharges

**DOI:** 10.3390/nano14010040

**Published:** 2023-12-22

**Authors:** Ye Rin Lee, Do Yeob Kim, Jae Young Kim, Da Hye Lee, Gyu Tae Bae, Hyojun Jang, Joo Young Park, Sunghoon Jung, Eun Young Jung, Choon-Sang Park, Hyung-Kun Lee, Heung-Sik Tae

**Affiliations:** 1 School of Electronic and Electrical Engineering, College of IT Engineering, Kyungpook National University, Daegu 41566, Republic of Korea; jane9991220@knu.ac.kr (Y.R.L.); jyk@knu.ac.kr (J.Y.K.); dlekgp1225@naver.com (D.H.L.); doctor047@knu.ac.kr (G.T.B.); bs00201@knu.ac.kr (H.J.); eyjung@knu.ac.kr (E.Y.J.); 2 Superintelligence Creative Research Laboratory, Electronics and Telecommunications Research Institute (ETRI), Daejeon 34129, Republic of Korea; nanodykim@etri.re.kr; 3 Department of Nano-Bio Convergence, Korea Institute of Materials Science, Changwon 51508, Republic of Korea; jypark@kims.re.kr (J.Y.P.); hypess@kims.re.kr (S.J.); 4 The Institute of Electronic Technology, College of IT Engineering, Kyungpook National University, Daegu 41566, Republic of Korea; 5 Department of Electrical Engineering, Milligan University, Johnson City, TN 37682, USA; cpark@milligan.edu

**Keywords:** air–liquid discharge, organic compound decomposition, pin–liquid barrier discharge, pin–liquid discharge, plasma processing, total dissolved phosphorus

## Abstract

To generate a stable and effective air–liquid discharge in an open atmosphere, we investigated the effect of the dielectric barrier on the discharge between the pin electrode and liquid surface in an atmospheric-pressure plasma reactor. The atmospheric-pressure plasma reactor used in this study was based on a pin–plate discharge structure, and a metal wire was used as a pin-type power electrode. A plate-type ground electrode was placed above and below the vessel to compare the pin–liquid discharge and pin–liquid barrier discharge (PLBD). The results indicated that the PLBD configuration utilizing the bottom of the vessel as a dielectric barrier outperformed the pin–liquid setup in terms of the discharge stability and that the concentration of reactive species was different in the two plasma modes. PLBD can be used as a digestion technique for determining the phosphorus concentration in natural water sources. The method for decomposing phosphorus compounds by employing PLBD exhibited excellent decomposition performance, similar to the performance of thermochemical digestion—an established conventional method for phosphorus detection in water. The PLBD structure can replace the conventional chemical-agent-based digestion method for determining the total dissolved phosphorus concentration using the ascorbic acid reduction method.

## 1. Introduction

Gas–liquid discharges, in which gaseous plasma directly contacts liquid materials and generates physical and chemical interactions, are not only useful in bacterial inactivation, sterilization, plant growth promotion, water treatment, material modification, and nanomaterial synthesis but also considered as a promising water treatment method and an advanced eco-friendly oxidation process [1,2,3,4,5,6]. To replace the conventional methods for decomposing and removing organic pollutants using chemical agents that may create secondary pollutants, research regarding the advanced oxidation process (AOP) is being actively conducted along with the emerging trend of sustainable green technology [7,8,9]. AOP involves the production of hydroxyl radicals (^•^OH), an intermediate species with a high redox potential (E^0^_•OH_ = 1.9–2.8 V vs. a normal hydrogen electrode), and their derivatives to nonselectively oxidize organic contaminants to less harmful intermediates [10]. The effective treatment of wastewater with ^•^OH-based AOP has been demonstrated by various research groups [11,12,13]. AOPs use photocatalysts, ozonation, plasma, subcritical water, and ultrasound instead of chemical agents, offering the advantage of a reagent-free and waste-free process [14,15]. Among these methods, plasma-assisted AOP is known to effectively decompose and remove various substances [16,17]. Recently, our research group has taken a keen interest in atmospheric-pressure (AP) plasma-assisted AOP. This method employs an air–liquid discharge to decompose phosphorus compounds to orthophosphate, holding promise for water-quality sensor applications [18,19].

Various atmospheric-pressure (AP) plasma generation methods have been developed to implement air–liquid discharges that can directly use nitrogen and oxygen molecules from the atmosphere as a source of reactive nitrogen and oxygen species generated by plasma [20,21,22]. Among them, pin–liquid discharge (PLD), which has a pin–plate electrode configuration and generates discharge when a liquid sample is placed on a plate electrode, is a very simple method to generate air–liquid plasma in an open atmosphere [22,23,24]. In PLD, the pin-type power electrode is advantageous for enhancing the local electric field and enabling the air discharge without any additional gas discharge. A metal plate that serves as a ground (GND) electrode is usually immersed in a liquid container to intensify the air–liquid plasma. Thus, an air plasma is directly produced between the pin-type metal electrode and liquid surface and interacts with the liquid molecules. During PLD, various physical and chemical interactions can occur inside and on the surface of the liquid owing to strong electric fields, UV radiation, thermal emission, energetic electrons, and abundant excited gaseous species and radicals [25]. Additionally, metal GND electrodes immersed in liquids can have additional effects on these physical and chemical interactions. Because the liquid vessel of a PLD reactor is usually made of glass, acryl, or polypropylene, a pin–liquid barrier discharge (PLBD) can also be implemented using the bottom of the vessel as a dielectric barrier [26]. Based on the investigation of the effective PLBD generation, the PLBD reactor must be further studied to study the plasma stability, plasma byproduct generation in liquids, and the suitability of PLBD for use in wastewater treatment.

The aim of this study was to induce a stable air–liquid discharge and effectively generate reactive species in a liquid medium. To achieve this, two air–liquid alternating-current (AC) discharge types—PLD and PLBD—were prepared by placing plate-type ground (GND) electrodes above and below the vessel of the AP plasma reactor. Deionized (DI) water was subjected to the two air–liquid discharge types to investigate their respective electrical and optical properties and compare the differences in the water-soluble reactive species produced by these air–liquid discharge types. To apply the air–liquid discharge as a phosphorus-compound digestion method for determining the phosphorus content in water, which is directly related to water-quality monitoring, an aqueous solution of phosphorus compounds and lake water were treated with the two air–liquid discharge types in an open atmosphere. This study also investigated the efficiencies of phosphorous digestion in laboratory-prepared samples and those collected from lake water. The results for these samples were compared using conventional thermochemical and AP plasma digestions.

## 2. Materials and Methods

### 2.1. Pin–Liquid Discharge and Pin–Liquid Barrier Discharge Systems

**Figure 1:**
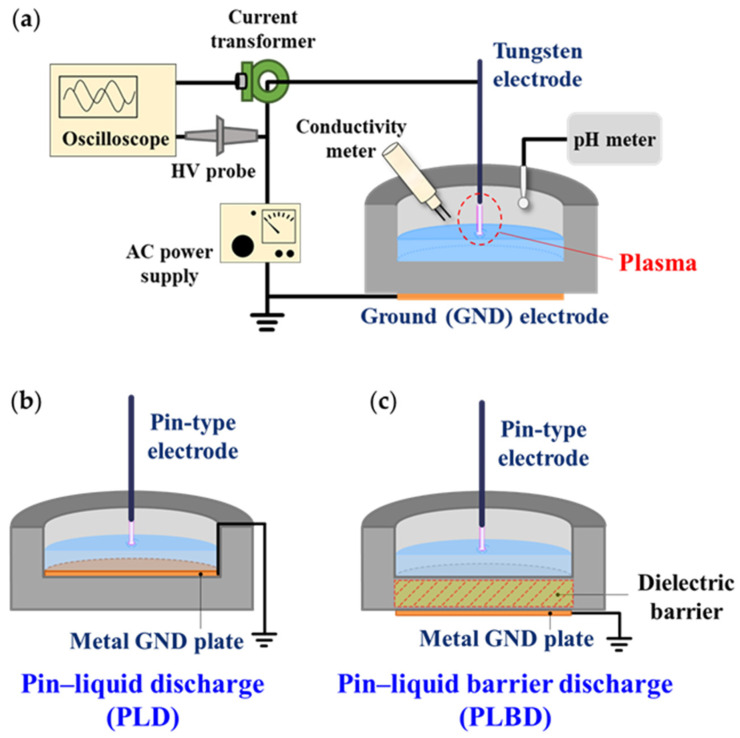
(**a**) Schematic diagram of the air–liquid discharge system consisting of a plasma device, a high-voltage (HV) power supply, and measurement instruments. Schematic diagrams of (**b**) PLD and (**c**) PLBD structures with GND plate electrodes at different positions.

The AP plasma system employed in the present study is depicted in Figure 1a. An inverter-type driving circuit was used to operate the AP plasma as it required a high voltage and a low current. To monitor the temporal electrical characteristics during the air–liquid discharge, the voltage and current waveforms were monitored using a high-voltage probe (P6015A, Tektronix Inc., Beaverton, OR, USA) and a current monitor (4100, Pearson Electronics Inc., Palo Alto, CA, USA), respectively. A compact spectrometer (USB-2000+, Ocean Optics Inc., Dunedin, FL, USA) equipped with an optical fiber was used to observe the excited species produced by the air plasma.

Figure 1b,c show the schematics of the PLD and PLBD reactors with a water vessel. A tungsten wire with a diameter of 500 μm was used as the pin-type power electrode. The cylindrical vessel containing the liquid was made of translucent polycarbonate with a permittivity (ε_r_) of 2.9. The height of the vessel was 26 mm, and the inner and outer diameters were 50 and 70 mm, respectively. The PLD and PLBD structures were prepared by placing copper disks with a diameter of 50 mm above and below the 10 mm thick bottom of the vessel, respectively, while keeping all the other design specifications the same. When this vessel was filled with 13 mL of water, the height of the water surface from the vessel bottom was 6.6 mm, and the spacing between the pin-type electrode and the water surface was 8 mm.

### 2.2. Measurement of Temperature, pH, and Conductivity

The conductivity of the plasma-irradiated water was measured using a pH/conductivity multiparameter meter (Orion Star A215, Thermo Fisher Scientific Inc., Waltham, MA, USA). The pH was measured in DI water using a pH meter (ST2100-F, Ohaus Corp., Parsippany, NJ, USA). The temperature of the plasma-irradiated water was measured by placing a thermocouple thermometer (D55, Hanyoung Nux, Incheon, Republic of Korea) in the water.

### 2.3. Measurement of Long-Lived Reactive Species (Hydrogen Peroxide, Nitrite, and Nitrate)

The concentration of hydrogen peroxide (H_2_O_2_) can be quantified by measuring that of the peroxovanadium cation (VO_3_^2+^), which is the product of the reaction among ammonium metavanadate (NH_4_VO_3_), sulfuric acid (H_2_SO_4_), and H_2_O_2_. Because the absorbance of VO_3_^2+^ is detected at a wavelength of ~450 nm, it does not overlap with the absorption spectra of conventional reactive nitrogen species; this is considered as a useful method for quantifying the H_2_O_2_ concentration without encountering interference from concurrent measurements [27]. The procedure entails combining 1.5 mL of 20 M NH_4_VO_3_ (Sigma-Aldrich Inc., St. Louis, MO, USA); 0.5 mL of H_2_SO_4_ (Sigma-Aldrich Inc., St. Louis, MO, USA); and 1.5 mL of H_2_O_2_ (Daejung Chemical & Metals Corp., Siheung, Republic of Korea) at concentrations spanning 0.01–10 mM. These mixtures were allowed to react for 3 min. A calibration curve was then obtained using the absorbance values of the mixed solutions with H_2_O_2_ concentrations of 0.01–10 mM by employing a compact spectrometer (USB-4000 UV–vis, Ocean Optics Inc., Dunedin, FL, USA). After performing linear fitting on the absorbances at the 450 nm wavelength for the various mixed solutions with different H_2_O_2_ concentrations, a high linearity of R^2^ = 0.998 was observed. Additionally, the trend line expressed as y = 0.0961x − 0.0192 (with y representing the absorbance optical density and x representing the concentration) was obtained. This calibration curve of H_2_O_2_ was used to quantify the H_2_O_2_ concentration from the absorbance measured at 450 nm in DI water treated using the PLD and PLBD types.

The nitrite (NO_2_^−^) and nitrate (NO_3_^−^) concentrations in the plasma-irradiated water were determined using a colorimetric assay. For this assay, colorimetric reagents (0.33 g of HI93708 for NO_2_^−^ and 0.22 g of HI93728 for NO_3_^−^; Hanna Instruments Inc., Woonsocket, RI, USA) were mixed with 10 mL of DI water treated with PLD and PLBD. The mixture was allowed to react for 10 min. The concentrations of NO_2_^−^ and NO_3_^−^ were quantified using an HI-97708 portable nitrite photometer and an HI-97728 portable nitrate photometer (Hanna Instruments Inc., Woonsocket, RI, USA), respectively. To quantify the concentration of NO_3_^−^, the plasma-treated water was diluted using DI water at a ratio of 2:3 (i.e., a 10 mL dilute solution comprised 4 mL of plasma-treated water and 6 mL of DI water). This dilution ensured that the resulting concentration remained below the upper detection limit of the nitrate photometer employed in this study. The results were validated by comparison with those for a standard nitrite solution (sodium nitrite; NaNO_2_) and a standard nitrate solution (potassium nitrate; KNO_3_), following the guidelines provided by the manufacturers.

### 2.4. Preparation of the Aqueous Phosphorus Compound and Lake Water Samples

The digestion performance of the two aqueous samples, i.e., organic phosphorus compound solution and lake water, using the air–liquid discharge was examined for determining the phosphorus content.

A solution of β-glycerol phosphate disodium salt pentahydrate (BGP; C_3_H_7_Na_2_O_6_P·5H_2_O, Sigma-Aldrich Inc., St. Louis, MO, USA) was prepared as an organic phosphorus compound sample. This solution had a phosphorus concentration of 1.0 mg/L and was prepared by dissolving 4.9 mg of BGP in 500 mL of DI water.

Samples of lake water were acquired from Lake Daecheong, the third largest lake in the Republic of Korea. Surface water samples were collected using thoroughly cleaned polypropylene containers (2.8 L) at two sampling points from Lake Daecheong. This artificial lake was created by blocking the water flow of the Geumgang River, situated in the suburban regions of Daejeon and Cheongju, Republic of Korea. The sampling was conducted in September 2022, and several physicochemical properties of the lake water were also measured using a multi-parameter water-quality sensor (YSI EXO2, YSI Inc., Yellow Springs, OH, USA). The detailed physicochemical properties of the lake water can be found in Table S1 (Supplementary Materials).

Following collection, the containers holding the lake water were immediately covered with aluminum foil and transported to the laboratory, where they were stored under low-temperature conditions to prevent degradation due to external light and heat. A microporous membrane with a pore size of 0.45 μm was used to prepare the samples for the subsequent analysis by removing suspended particles, including fine residues, colloidal components, and biological microorganisms, via filtration. The filtration processes were conducted at a pressure of 0.2 bar, serving as a precursor to the plasma treatment.

The BGP solution and lake water samples were each introduced to the vessels of the PLD and PLBD reactors at volumes of 13 mL. Subsequently, these samples were subjected to air-plasma treatment for 1–20 min.

For the thermochemical digestion of the lake water, a sequence of steps was followed. After the filtration process, a mixture comprising 50 mL of the lake water and 10 mL of a 4% potassium persulfate (K_2_S_2_O_8_; Sigma-Aldrich Inc., St. Louis, MO, USA) solution was prepared and sealed in a Teflon bottle. Then, the mixture was heated at 120 °C for 21 min using an electric oven. Subsequently, the mixture was allowed to cool naturally to room temperature, resulting in an overall time requirement of 4 h, including the heating and cooling stages.

### 2.5. Ascorbic Acid Reduction Method

Different amounts of potassium phosphate monobasic (KH_2_PO_4_, Sigma-Aldrich Inc., St. Louis, MO, USA) were dissolved in DI water to prepare phosphate standard solutions containing 0.20, 0.40, 0.60, 0.80, and 1.0 mg/L of phosphorus. For the ascorbic acid reduction method, 0.18 g of a colorimetric reagent (HI713-25, Hanna Instruments Inc., Woonsocket, RI, USA) was mixed and reacted with 10 mL of each standard solution for 10 min. Subsequently, a calibration curve was established using the absorbance values of the phosphate standard solutions at a wavelength of 710 nm, as obtained using a compact spectrometer (USB-4000 UV–vis, Ocean Optics Inc., Dunedin, FL, USA) [28]. The decomposition of the phosphorus compounds to orthophosphate in the BGP solution and lake water, after the plasma treatment or thermochemical digestion, was identified by comparing the calibration curve with various concentrations derived from the phosphate standard solutions.

### 2.6. Ultraviolet–Visible Absorption Spectral Measurements

The absorption spectra of the processed solutions were measured using an ultraviolet–visible (UV–vis) spectrophotometer (LAMBDA 950, Perkin Elmer Inc., Waltham, MA, USA) at the Korea Basic Science Institute (Daegu, Korea) and quartz cuvette in a wavelength range of 200–1000 nm.

### 2.7. Statistical Analysis

All the quantitative data for the plasma-treated water are expressed as the mean ± the standard deviation (SD) for each experiment in three replicates (n = 3).

## 3. Results and Discussion

### 3.1. Structural Features of Pin–Liquid Discharge and Pin–Liquid Barrier Discharge Reactors

The PLBD reactor differs from the PLD reactor only in the placement of the metal plate below the vessel. This metal plate serves to introduce a dielectric barrier to the discharge path (Figure 1b,c). From the viewpoint of plasma–liquid interactions, it is important to note that neither electrode comes into direct contact with the liquid, which is an important structural feature of the PLBD. This electrode configuration does not allow charged particles generated via the liquid during the gas–liquid discharge to escape through the metal electrode. Moreover, the plasma-treated solution avoids reacting with the metal electrode, preventing the formation of metal-ion-related byproducts. The PLD reactor has fundamentally the same configuration as a contact glow-discharge electrolysis device, where one electrode is completely immersed in the solution [29]. Hence, PLD involves a combination of gaseous glow-discharge and liquid electrolysis processes, yielding different experimental outcomes that can be distinguished from those achieved with PLBD for water treatment through plasma. Contact glow-discharge electrolysis generally requires a high DC voltage. However, for the current experiment, both discharge reactors were operated using a sinusoidal AC voltage. Despite this adaptation, contact glow-discharge electrolysis may still occur during the negative half-cycle of the applied AC voltage in the PLD process, causing a difference in the reactive species generated in the water through PLD and PLBD.

### 3.2. Discharge Properties of Pin–Liquid Discharge and Pin–Liquid Barrier Discharge

**Figure 2:**
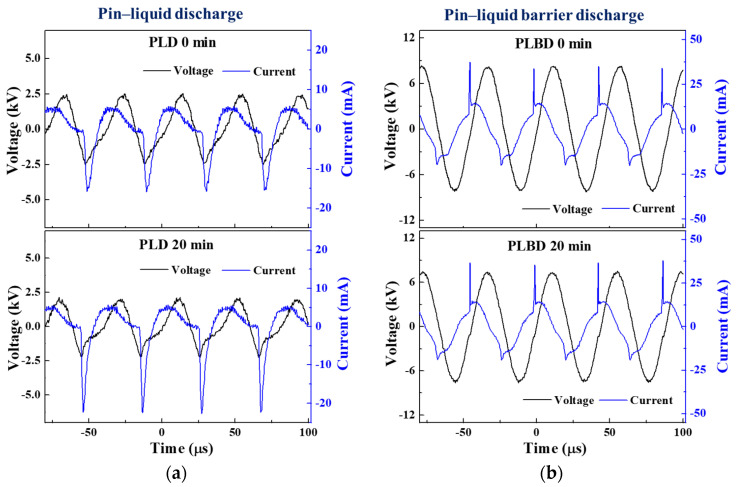
AC voltage and current waveforms measured at the beginning (0 min) and after 20 min of (**a**) PLD and (**b**) PLBD under optimal operating conditions.

Using the bottom of the reactor vessel as a dielectric barrier during the air–water discharge has the advantage of obtaining a stable glow plasma because the discharge current can be effectively controlled over time. When driving the PLD and PLBD reactors using an AC sinusoidal voltage, disparities inevitably arise in the operating conditions of the two discharge methodologies. These disparities are attributed to the differences in the electrode structure and discharge procedures. Following a series of trial runs, the optimal operation conditions for the PLD and PLBD methods were ascertained. A sinusoidal voltage with peak values of 2.7 and 7.5 kV was determined for the PLD and PLBD methods, respectively. Notably, these settings ensured the generation of a stable and efficient discharge. Both applied sinusoidal voltages shared a frequency of 22 kHz. Figure 2a,b show the waveforms of the applied voltage and the corresponding current measured for four cycles during 20 min of operation for PLD and PLBD, respectively. The input power is calculated according to Equation (1) as follows:

(1) $$P=\frac{1}{T}\int_{0}^{T}U\left(t\right)\times I\left(t\right)dt$$

where T is the period of the applied voltage, U(t) is the voltage signal, I(t) is the acquired current, and t is the time, respectively. For the PLD and PLBD methods, the input power was calculated at 5.93 W and 10.36 W at the beginning (0 min) and 4.60 W and 9.82 W at 20 min of processing, respectively.

As shown in Figure 2a, the discharge occurred mainly during the negative half-cycle of the applied voltage in the PLD mode. When PLD occurred, the current waveform appeared distorted because it contained the conduction current flowing in the water medium, and the discharge current flowed through the air between the pin electrode and the water surface. In Figure 2b, the current waveform for the PLBD mode shows a swift spike in the discharge current over a short period. Additionally, a sinusoidal displacement current appeared owing to the charging and discharging cycles of the dielectric barrier. Because the dielectric barrier served as a capacitive element, a phase difference was also observed between the applied voltage and the sinusoidal displacement current. The current waveform of the PLBD showed that discharge occurred during the rising phase and the falling phase of the voltage waveform. A notably more intense discharge current was observed during the positive half-cycle of the applied voltage, when the pin electrode acted as an anode.

In the PLD mode, the sinusoidal voltage waveform became more distorted, and the current waveform increased in the negative half-cycle after 20 min of air–water discharge, in contrast to the initial voltage and current waveforms (depicted in the upper and lower graphs of Figure 2a). During PLD, a plasma column initiated within the air, establishing electrical connectivity between the power electrode and water surface and rendering the water in the reactor conductive. Given that the plate GND electrode maintained direct contact with the water in the PLD mode, the two electrodes were electrically connected through the path of the plasma column and conductive water, allowing a conduction current to flow between them. As the conductivity of the water progressively increased within the reactor owing to the air–water discharge, the current magnitude was noted to escalate during the negative half-cycle of the voltage waveform, primarily after 20 min of air–water discharge.

Meanwhile, in the case of PLBD, where neither electrode made contact with the water, the conduction current related to the conductivity of the water remained absent during the air–water discharge (as shown in the upper and lower graphs of Figure 2b). Therefore, the current waveform, comprising the displacement current due to the charge/discharge of the dielectric barrier and the discharge current due to the plasma generation, exhibited minimal alterations, except for the slightly reduced amplitude of the voltage waveform, even after 20 min.

**Figure 3:**
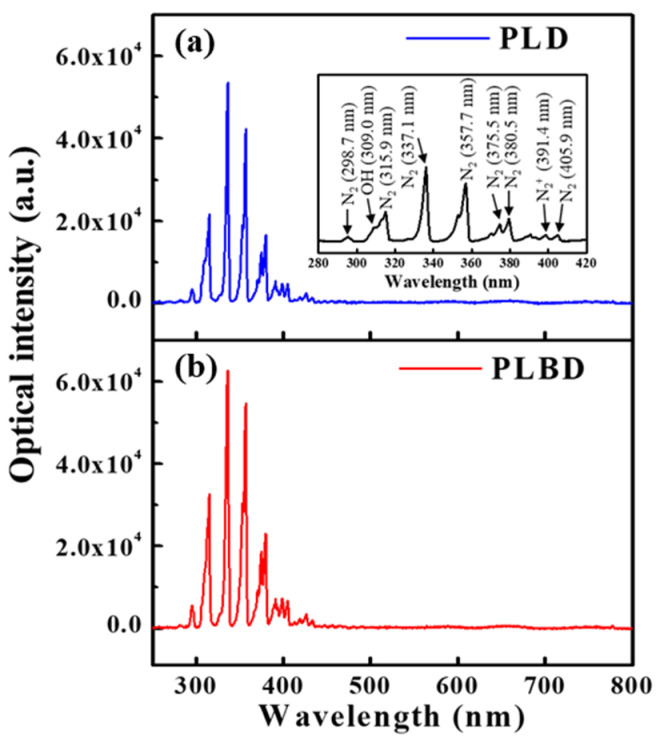
Optical emission spectra measured in air during (**a**) PLD and (**b**) PLBD.

Optical emission spectra (OES) were obtained to identify the excited species generated in the gaseous phase during both PLD and PLBD. The emission spectra from 250 to 800 nm during PLD (Figure 3a) and PLBD (Figure 3b) revealed the presence of excited N_2_, N_2_^+^, and ^•^OH species, which were generated as a consequence of the air–water discharge (Figure 3a). Notably, these excited species originated from the atmosphere as the air–water discharge process did not involve the use of additional discharge gases, such as argon or helium.

**Figure 4:**
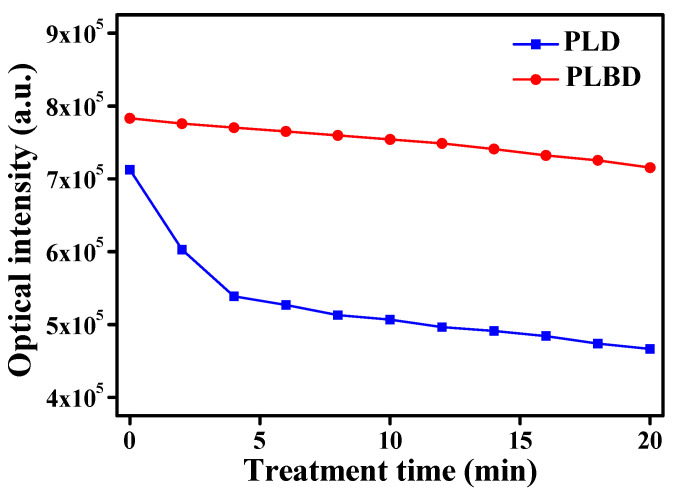
Changes in the optical intensity of the glow emission in PLD and PLBD measured at 2 min intervals for 20 min.

The alterations in the optical intensity of the glow emission at 2 min intervals during 20 min of operation for both PLD and PLBD are presented in Figure 4. The initial discharge (0 min) showed a higher total peak intensity for PLBD in the OES data than for PLD, which is attributed to the higher voltage operation of PLBD. Over the 20 min period, the optical emission in the PLD mode decreased by 35%, whereas the decrease was approximately 9% in the PLBD mode. This discrepancy can be attributed to the variations in the water’s conductivity. The flow of the conduction current between the electrodes is promoted at the same applied voltage as the water’s conductivity gradually increases. This behavior can also be observed in the voltage and current waveforms, as shown in Figure 2a. After 20 min of the PLD treatment, it is observed that the voltage slightly decreases and the current peak increases compared to those during the initial plasma treatment. These electrical properties indicate a decrease in the load impedance of the plasma medium. The increase in the water’s conductivity reduces the effect of the load impedance on the electrical equivalent circuit of the air–liquid plasma system, which is observed as a reduction in the optical emission of the glow plasma in air. Consequently, the observed reduction emission intensity was more prominent in the PLD mode, primarily influenced by the water’s conductivity. In contrast, PLBD demonstrated an advantage in maintaining a relatively stable air–liquid discharge over time. Notably, the rise in the water temperature and the evaporation of the DI water subjected to both PLD and PLBD for 20 min did not substantially impact the water activation and digestion results, as detailed in Section S1 and Figure S1 (Supplementary Materials).

### 3.3. Assessment of the Long-Lived Reactive Species Formed through Plasma–Water Interaction

A portion of the reactive species and radicals generated via the gas discharge can dissolve in water or interact with water molecules, thereby generating aqueous reactive species [30]. Several studies have reported the presence of elevated levels of reactive oxygen and nitrogen species (RONS) in plasma-activated water (PAW) [30,31,32]. Studies on the reactivity of PAW have investigated the presence of diverse reactive chemical species in water, including ^•^OH, H_2_O_2_, ozone (O_3_), superoxide (O_2_^−^), nitrogen oxides (NO_x_), and peroxynitrite (HONO_2_) [33]. Owing to the presence of these RONS, PAW exhibits a distinctive electrical conductivity and acidity. Because of the wide variety of reactive species that can coexist in water during air–water discharges, pinpointing the precise ions contributing to the increase in the electrical conductivity proves challenging. Nevertheless, prior research has evidenced a correlation between the low pH of plasma-treated water samples and a high electrical conductivity [30,34].

**Figure 5:**
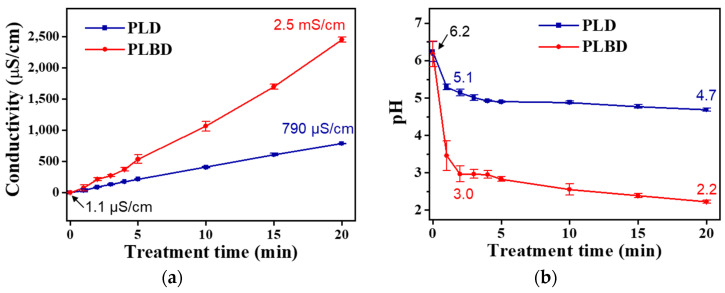
Temporal changes in (**a**) electrical conductivity and (**b**) pH of DI water treated through PLD and PLBD. Data are presented as the mean ± SD of three repeated experiments.

Figure 5 depicts the temporal changes in the electrical conductivity and pH of DI water treated with the PLD and PLBD reactors. The electrical conductivity of the plasma-irradiated water increased with prolonged plasma exposure across both discharge types; however, this increase was greater in PLBD. In the context of the DI water treated with PLBD for 20 min, the electrical conductivity surged from 1.1 μS/cm to 2.5 mS/cm (Figure 5a), surpassing the conductivity of 790 μS/cm observed for DI water treated with PLD for the same duration. The hydrogen-ion concentration, as reflected by the pH values, swiftly declined, transitioning to an acidic range within the initial 2 min across both discharge cases. This rapid pH shift stemmed from the prompt generation of H^+^ via the interaction between the water molecules and air–water discharge (Figure 5b). Evidently, compared with its PLD-treated water counterpart, the PLBD-treated water exhibited a more rapid descent in pH with increasing plasma irradiation time. Specifically, for the PLBD-treated water, the pH rapidly decreased from 6.2 to 3.0 in the initial 2 min, followed by a relatively gradual decline from 3.0 to 2.2 over the subsequent 18 min. Following a 20 min PLBD, the resulting solution exhibited a pH of 2.2, which was much lower than the pH recorded after 20 min of PLD (4.7), confirming the high acidity of the PLBD-treated water.

As shown in Figure 5, the electrical conductivity for the PLBD case surpassed that for the PLD case, particularly when the pH of the DI water treated through PLBD was lower than that of the DI water treated through PLD. Notably, the pH and electrical conductivity of liquid samples are influenced by various plasma parameters, such as the discharge type, electrode position, applied voltage and frequency, treatment time, and sample volume [35,36]. Based on the voltage and current waveforms illustrated in Figure 2, which show that PLBD generates a more intense plasma than PLD, it is reasonable to infer that the DI water subjected to PLBD would exhibit a lower pH and higher electrical conductivity than that subjected to PLD. Furthermore, as described later, the decomposition of H_2_O_2_ to water and oxygen in the PLD mode maintains a high pH, potentially influencing the electrical conductivity in an unfavorable manner.

The interaction among the energetic electrons, excited ions, and radicals generated by the air–water discharge led to the formation of aqueous reactive species through their interactions with water molecules. Alongside short-lived reactive species, long-lasting counterparts, such as H_2_O_2_, NO_2_^−^, and NO_3_^−^, were also detected in the plasma-treated water. These long-lived reactive species persist in the water for extended periods, ranging from tens of minutes to several days, effectively activating the water at a chemical level [37]. The quantitative analysis of the long-lived species, including H_2_O_2_, NO_2_^−^, and NO_3_^−^, serves as solid evidence to assess water activation though plasma treatment [38,39]. Consequently, measurements of the concentrations of H_2_O_2_, NO_2_^−^, and NO_3_^−^ in both PLD- and PLBD-treated DI water were performed to comprehensively investigate the water activation achieved through both discharge types.

**Figure 6:**
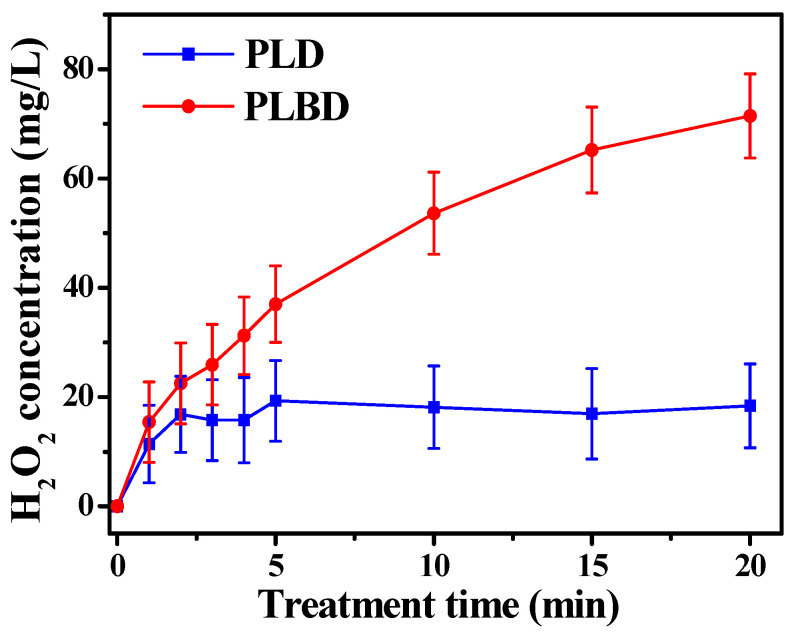
Concentration of H_2_O_2_ in plasma-treated DI water as a function of plasma treatment time for PLD and PLBD. Data are presented as the mean ± SD of three repeated experiments.

Figure 6 depicts the variations in the H_2_O_2_ concentration in the DI water as a function of the treatment time for both discharge types. It is known that ^•^OH generated through air discharges undergoes recombination to form H_2_O_2_ [38]. As shown in Figure 6, a distinct difference between the H_2_O_2_ concentrations of the two discharge types was observed as the treatment time progressed. Specifically, the H_2_O_2_ concentration in the PLD-treated water reached saturation at approximately 20 mg/L within 5 min and remained relatively constant thereafter. Meanwhile, the H_2_O_2_ concentration in the PLBD-treated water continued to increase throughout the 20 min period. Following 20 min of plasma treatment, the H_2_O_2_ concentration in the PLBD case surpassed that in the PLD case by a factor of nearly 3.5.

**Figure 7:**
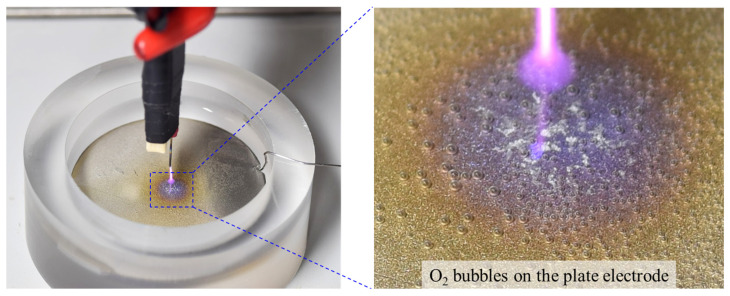
Oxygen bubbles observed on the titanium plate electrode submerged in the DI water during PLD. A photograph of the PLD reactor and its enlarged image.

The considerable difference between the H_2_O_2_ concentrations observed for the two discharge types can be attributed to the H_2_O_2_ loss incurred in the water electrolysis process during PLD. As previously mentioned, the proposed PLD reactor shares the electrode configuration of contact glow-discharge electrolysis, allowing for both air-based glow discharge and water-based electrolysis. Hence, the H_2_O_2_ generated during PLD is simultaneously decomposed to oxygen and water through electrolysis, resulting in a partial reduction of the resultant H_2_O_2_. As the production and depletion of H_2_O_2_ reached equilibrium, the H_2_O_2_ concentration no longer increased. Moreover, the electrolysis-induced H_2_O_2_ loss accounted for the higher liquid pH observed in PLD than in PLBD (Figure 5b). The electrolytic conversion of H_2_O_2_ to oxygen and water during PLD can be visually confirmed by observing transparent bubbles forming on the titanium plate electrode submerged in the DI water (Figure 7).

**Figure 8:**
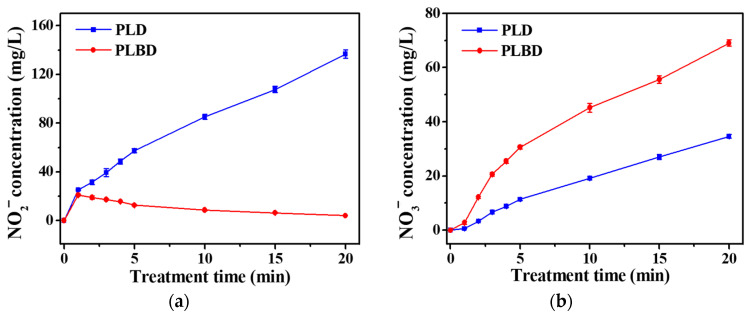
Temporal changes in (**a**) nitrite (NO_2_^−^) and (**b**) nitrate (NO_3_^−^) concentrations in DI water treated through PLD and PLBD. Data are presented as the mean ± SD of three repeated experiments.

Figure 8 depicts the variations in the NO_2_^−^ and NO_3_^−^ concentrations in the DI water with increasing treatment time for both PLD and PLBD. As shown in Figure 8a, the NO_2_^−^ concentration in the PLD case exhibited a linear increase with increasing treatment time, reaching approximately 135 mg/L after 20 min. However, PLBD led to an initial peak in the NO_2_^−^ concentration of 20 mg/L after just 1 min, which subsequently exhibited a gradual decline. In air plasma, nitric oxide (NO) is primarily produced through the reactions outlined in Equations (2) and (3), known as the Zel’dovich mechanism [40]. The resultant NO can be easily oxidized to NO_2_ in the gaseous plasma medium through the reactions given by Equations (4) and (5) [41].

O + N_2_ → NO + N (2)

N + O_2_ → NO + O (3)

O + NO + M → NO_2_ + M, where M = N_2_, O_2_, NO, and NO_2_ (4)

NO + O_2_ → NO_2_ + O_2_ (5)

The formation of NO_2_^−^ and NO_3_^−^ in water is the result of the dissolution of NO and NO_2_ formed in air plasma, respectively. When generated NO and NO_2_ dissolve in water, they produce NO_3_^−^ and NO_2_^−^ as well as H^+^ ions, which lower the solution pH, as shown in Equations (6) and (7), respectively [42].

NO_2_ + NO_2_ + H_2_O → NO_2_^−^ + NO_3_^−^ + 2H^+^ (6)

NO + NO_2_ + H_2_O → 2 NO_2_^−^ + 2H^+^ (7)

An additional pathway for the depletion of NO_2_^−^ and production of NO_3_^−^ is followed when NO_2_^−^ reacts with H_2_O_2_, leading to the formation of peroxynitrous acid (HONO_2_), particularly under highly acidic conditions. This intermediate species is known to be unstable; it rapidly decomposes to the final product, NO_3_^−^, as shown in Equations (8) and (9).

NO_2_^−^ + H_2_O_2_ + H^+^ → HONO_2_ + H_2_O (8)

HONO_2_ → NO_3_^−^ + H^+^ (9)

Therefore, in the PLBD-treated water, at a reduced pH, the measured NO_2_^−^ concentration was considerably low (Figure 8a), while the measured NO_3_^−^ concentration exceeded that in the PLD-treated water (Figure 8b). Additionally, PLBD at a higher plasma intensity compared with that of PLD (Figure 4) could promote the formation of gaseous NO_2_, leading to higher NO_3_^−^ concentrations in the PLBD-treated water [43]. As depicted in Figure 8b, the NO_3_^−^ concentrations in the DI water subjected to both discharge methods increased over time; however, the NO_3_^−^ concentration following 20 min of air–water discharge through PLBD was twice of that achieved with PLD, as previously mentioned.

### 3.4. Decomposition of Phosphorus Compounds in Aqueous Solutions

Recently, various studies related to the treatment of organic compounds using a plasma-based advanced oxidation process have been reported [44,45]. Among them, the decomposition of phosphorus compounds in water via plasma technology, which is important in designing water-quality sensors and the prediction of harmful algal blooms, has caught our attention. Because phosphorus enters the freshwater aquatic environment through various pathways, it exists in the form of various compounds that make its detection and monitoring a challenge. Therefore, the total phosphorus concentration is generally measured after the diverse phosphorus compounds are digested to form orthophosphate, the primary soluble form of phosphate. There are several analysis methods that use fluorescence emission spectra [46] and diffusive gradients in thin films [47] for the highly sensitive detection of pollutant concentrations in water. However, in this study, the ascorbic acid reduction method, the most used method for quantifying orthophosphate according to the water-quality standards of the U.S. Environmental Protection Agency, is used to determine the amount of orthophosphate digested from various phosphorus compounds dissolved in freshwater [48,49].

As an important application of the air–liquid discharge, we focused on the decomposition of aqueous phosphorus compounds. The proposed PLD and PLBD methods, which are types of plasma-assisted AOP, also use ^•^OH and its derivatives generated by AP plasma between air and water as oxidizing agents to digest phosphorus compounds. When AP plasma is produced near water, some of the generated reactive species can dissolve in the water or interact with water molecules to generate a variety of aqueous reactive species. Among the generated aqueous reactive species is ^•^OH, which is known to be the most reactive oxidizing agent in water treatment and can be used to decompose phosphorus compounds in water [10,18].

**Figure 9:**
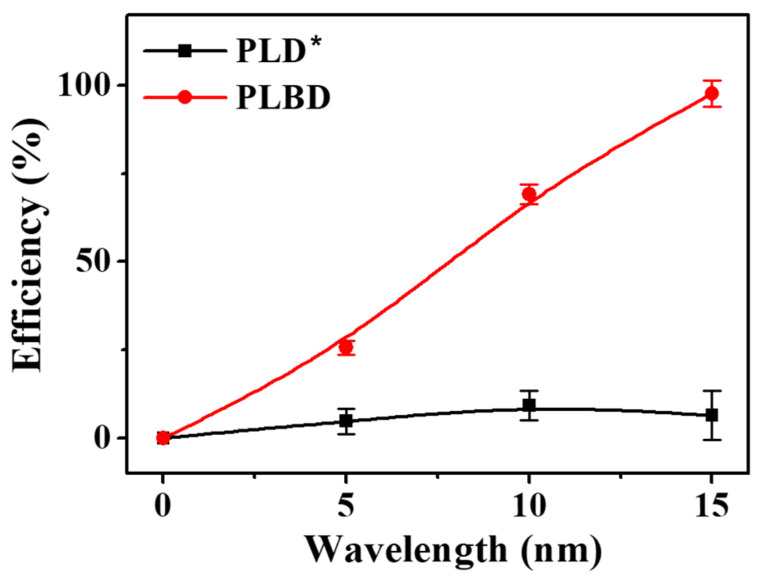
Temporal changes in the efficiency of BGP decomposition to orthophosphate via PLD and PLBD (mean ± SD of triplicate experiments). * For PLD, the BGP decomposition efficiency can be underestimated.

A 13 mL BGP solution, containing 1.0 mg/L of phosphorus, was prepared and subjected to both PLD and PLBD for 15 min. The efficiencies of the decomposition for both discharge methods, as assessed using the ascorbic acid reduction method, are depicted in Figure 9. The decomposition efficiency of PLBD reached 97.7% in a 15 min period, indicating that the majority of the BGP in solution was converted to orthophosphate. In contrast, the PLD-treated BGP solution exhibited a considerably lower decomposition efficiency, remaining <10%, even with extended plasma-treatment exposure, suggesting the inadequate performance of the ascorbic acid reduction method in PLD. This disparity is attributed to the direct contact of the solution sample with the metal-plate-type electrode during PLD. Ascorbic acid, known for its high redox activity, readily reacts with metal ions present in solutions, leading to oxidation [50]. The electrolysis-induced release of metal ions from the plate electrode during PLD can substantially interfere with the efficacy of the ascorbic acid reduction method [51].

**Figure 10:**
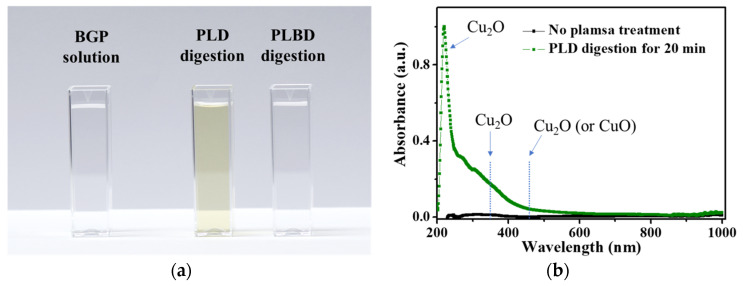
(**a**) Photograph of untreated, PLD-treated, and PLBD-treated BGP solutions and (**b**) UV–vis spectra of the untreated BGP solution and BGP solution treated with PLD for 30 min.

When examining the plasma-treated BGP solution using the PLD reactor with a copper plate, it was observed that copper ions were released from the copper plate through contact glow-discharge electrolysis, producing copper oxide nanoparticles in the BGP solution (Figure 10a). The UV–vis absorption spectrum of the PLD-treated BGP solution showed that the resulting nanoparticles were mainly cuprous oxide (Cu_2_O), as shown in Figure 10b [52,53]. Efforts to mitigate the interference with the efficacy of the ascorbic acid-reduction method by the metal-ion release, such as by changing the electrode material from copper to iron, titanium, or tungsten, did not yield positive results. Consequently, the decomposition of phosphorous compounds via PLD cannot be considered as a feasible digestion process for the ascorbic acid reduction method owing to this interference.

**Figure 11:**
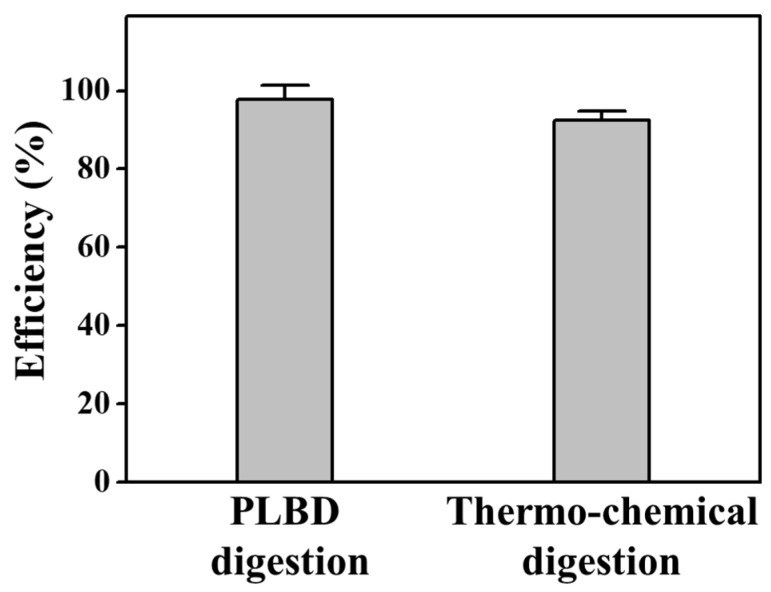
Decomposition efficiency for orthophosphate during PLBD and thermochemical digestions (mean ± SD of triplicate experiments).

In Figure 11, a comparison is presented for the decomposition efficiencies of the BGP solution treated with thermochemical and PLBD digestions for 15 min. The thermochemical digestion of the BGP solution resulted in an efficiency of 92.3% for conversion to orthophosphates. However, the proposed PLBD digestion outperformed this, achieving an even higher efficiency of 97.7%. This heightened efficiency positions PLBD digestion as a more potent method. Consequently, this decomposition approach utilizing PLBD holds promise as an effective digestion process for the quantification of the phosphorus concentration in water, a key indicator of the water quality in rivers and lakes.

Table 1 presents the total dissolved-phosphorus concentrations measured in the lake water sourced from Lake Daecheong, Republic of Korea. The analysis was conducted using the ascorbic acid reduction method with both thermochemical and PLBD digestions. The thermochemical digestion, requiring approximately 4 h, yielded a total dissolved-phosphorus content of 66.3 ppb (0.0663 mg/L). The PLBD digestion, however, performed for only 20 min, resulted in a total dissolved-phosphorus content of 68.1 ppb (0.0681 mg/L). These findings highlight the superiority of PLBD digestion over conventional techniques. Additionally, PLBD digestion using air–water plasma showcases environmental friendliness as it omits the need for chemicals, such as potassium persulfate. Compared with thermochemical digestion, which demands a high-temperature and -pressure reaction chamber, PLBD digestion also offers advantages, such as rapid analysis and a downsized water-quality sensor system. Given the comprehensive assessment encompassing the digestion performance, usability, processing costs and time, eco-friendliness, and on-site real-time applicability, the proposed plasma digestion emerges as a feasible alternative technology to replace existing phosphorus-compound digestion methods.

## 4. Conclusions

This study explored the discharge characteristics of two gas–liquid discharge treatments—PLD and PLBD, based on the pin–plate electrode configuration. The research also explored the impacts of the dielectric barrier on DI water activation and the digestion of phosphorus compounds during the air–liquid discharge. The analysis showed notable differences between PLD and PLBD for water treatment owing to the absence or presence of a dielectric barrier along the air–water discharge path. In PLD, where the plate electrode was submerged in water, electrolysis played a pivotal role, leading to distinctive outcomes compared with those for PLBD. Both PLD and PLBD led to the generation of long-lived reactive species that effectively activated DI water. However, the quantities of the reactive species produced using these methods demonstrated different trends with increasing plasma-treatment duration. In the case of PLD, the continuous electrolysis from H_2_O_2_ to water and oxygen resulted in the limited contribution of H_2_O_2_ to water activation. However, in PLBD, the production of HONO_2_ in low-pH environments consumed NO_2_^−^ to produce NO_3_^−^. This led to a lower level of NO_2_^−^ compared with that of NO_3_^−^ in PLBD. The proposed PLBD treatment is not desirable for producing a large amount of PAW in a short time owing to the small area of the gaseous plasma medium in contact with the water. However, as a result of this study, it can be inferred that treating water with DBD is advantageous for ^•^OH production because contact glow-discharge electrolysis is avoided, even if the design of the plasma reactor is changed to increase the area in contact with the water for the mass production of PAW. Additionally, a practical application for the air–water discharge was demonstrated through the digestion of phosphorus compounds in aqueous solutions, transforming the compounds to simpler substances, such as orthophosphate. Although PLD digestion introduced complications by releasing aqueous metal ions from the submerged plate electrode, which interfered with the ascorbic acid reduction method, the performance of the PLBD digestion aligned well with that of the conventional thermochemical digestion without affecting colorimetric reactions. Consequently, PLBD demonstrated its capability for efficiently digesting phosphorus compounds in <20 min, suggesting its applicability for in situ, real-time measurements of total dissolved-phosphorus concentrations in water, using the ascorbic acid reduction method. The findings from the digestion experiments using lake water revealed the potential of PLBD as a promising alternative to sample digestion for phosphorus concentration determination, enabling the prediction of harmful algal blooms in rivers and lakes. This research offers insights into enhancing water-quality assessment and environmental monitoring methodologies.

## Figures and Tables

**Table 1:** Comparison of total dissolved-phosphorus concentrations detected in real water samples through thermochemical and PLBD digestion methods.

Sample	Digestion Method	Concentration (μg/L) (Mean ± SD, n = 3)
Lake water (Lake Daecheong)	Thermochemical	66.3 ± 2.52
	PLBD	68.1 ± 3.24

## Data Availability

Data are contained within the article and supplementary materials.

## Supplementary Materials

The following supporting information can be downloaded at https://www.mdpi.com/article/10.3390/nano14010040/s1, Figure S1: (a) Temperature change of DI water during PLD and PLBD treatments. (b) Volume change of DI water due to evaporation during air–water discharge. Data are presented as the mean ± SD of three replicates; Table S1: Physicochemical characteristics of the water sample from Lake Daecheong.
