# Supplementary material for: Effects of Dielectric Barrier on Water Activation and Phosphorus Compound Digestion in Gas–Liquid Discharges
Source: Nanomaterials (Basel). 2023 Dec 22;14(1):40. doi: 10.3390/nano14010040 (PMC10780582; doi:10.3390/nano14010040)
Supplement: Supplementary file 1 [file nanomaterials-14-00040-s001.zip › nanomaterials-2745370-supplementary.pdf]

S1. Changes in water temperature and evaporation volume of DI water treated with both PLD and PLBD

Fig. S1a shows the temperature changes for the DI water treated with both the PLD and PLBD air–water discharges. The temperature of the DI water was measured by placing a thermocouple thermometer (D55, Hanyoung Nux, Incheon, South Korea) in the water. In both discharge treatments, the temperature of the DI water rapidly increased above 20 °C during the first 5 min of the plasma treatments and then gradually increased until 20 min, reaching saturation at around 50 °C. Because the PLD reactor has no dielectric barrier, when the DI water becomes conductive, a conduction current can flow through the water medium, resulting in Joule heating.

During Joule heating by the conduction current, it was observed that the temperature of the PLD-treated DI water was slightly higher than that of the PLBD-treated DI water in all the regions and for all the treatment times, despite the input power of PLD being lower than that of PLBD. However, the difference was not considerable up to 5 °C. From the temperature monitoring, it was observed that even when the DI water was irradiated with both discharge methods for 20 min, the water temperature did not rise above 50 °C and was saturated, suggesting that the heat produced by the air–water discharge was low enough to have hardly any effect on the generation of aqueous reactive species. Fig. S1b shows the volume of the DI water lost by evaporation in both plasma treatments. Slightly more water evaporated during PLD owing to the higher temperature, but merely 2.5 mL of the sample evaporated, even after 20 min of plasma treatment. Therefore, sample evaporation using plasma treatment has little effect on the quantification of the phosphate concentration.

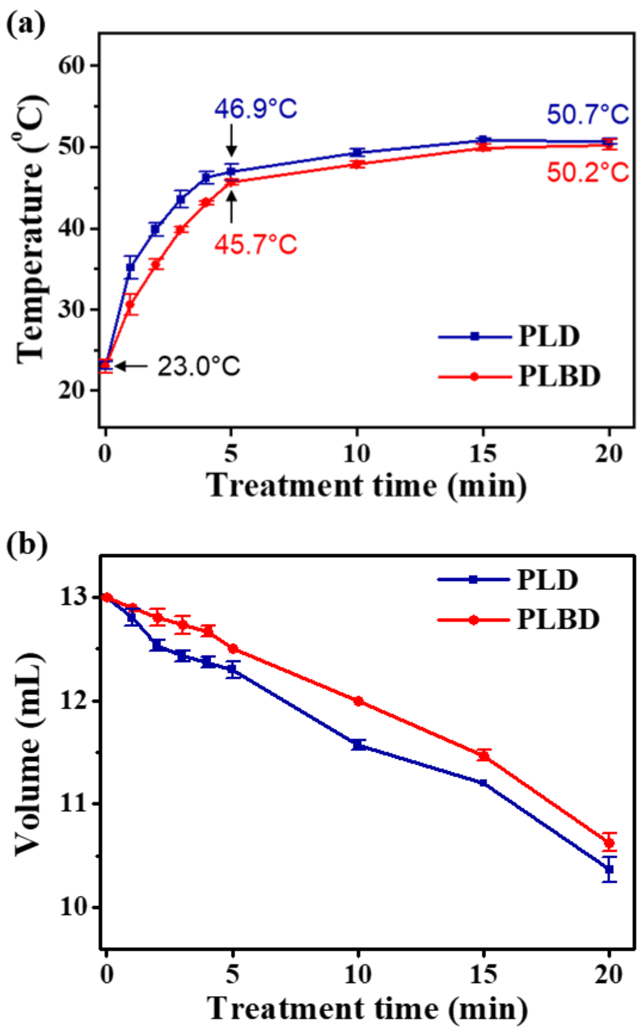

Figure S1. (a) Temperature change of DI water during PLD and PLBD treatments. (b) Volume change of DI water due to evaporation during air–water discharge. Data are presented as the mean ± SD of three replicates.

Table S1. Physicochemical characteristics of the water sample from Lake Daecheong.

Sampling Date: September 16, 2022.

Parameter	Value
Temperature	26.2 °C
pH	9.6
Turbidity	17.2 FNU
Conductivity	177.4 μS/cm
Chlorophyll concentration	7.1 μg/L
Phycocyanin (BGA-PC) concentration	3.8 μg/L
Dissolved oxygen concentration	13.7 mg/L
